# Factors impacting the regulation of *nos* gene expression in *Staphylococcus aureus*

**DOI:** 10.1128/spectrum.01688-23

**Published:** 2023-09-25

**Authors:** Jessica N. Brandwein, Tiffany S. Sculthorpe, Miranda J. Ridder, Jeffrey L. Bose, Kelly C. Rice

**Affiliations:** 1 Department of Microbiology & Cell Science, Institute of Food and Agricultural Sciences, University of Florida, Gainesville, Florida, USA; 2 Department of Microbiology, Molecular Genetics and Immunology, University of Kansas Medical Center, Kansas City, Kansas, USA; University of Minnesota Twin Cities, St. Paul, Minnesota, USA

**Keywords:** *Staphylococcus aureus*, bacterial nitric oxide synthase, β-galactosidase reporter, SrrAB, Agr, gene regulation

## Abstract

*Staphylococcus aureus* nitric oxide synthase (saNOS) contributes to oxidative stress resistance, antibiotic tolerance, virulence, and modulation of aerobic and nitrate-based cellular respiration. Despite its involvement in these essential processes, the genetic regulation of *nos* expression has not been well characterized. 5′ rapid amplification of cDNA ends on *nos* RNA isolated from *S. aureus* UAMS-1 (USA200 strain) and AH1263 (USA300 strain) revealed that the *nos* transcriptional start site mapped to an adenine nucleotide in the predicted Shine-Dalgarno site located 11 bp upstream of the *nos* ATG start codon, suggesting that the *nos* transcript may have a leaderless organization or may be subject to processing. The SrrAB two-component system (TCS) was previously identified as a positive regulator of *nos* RNA levels, and experiments using a β-galactosidase reporter plasmid confirmed that SrrAB is a positive regulator of *nos* promoter activity. In addition, the quorum-sensing system Agr was identified as a negative regulator of low-oxygen *nos* expression in UAMS-1, with activity epistatic to SrrAB. Involvement of Agr was strain dependent, as *nos* expression remained unchanged in an AH1263 *agr* mutant, which has higher Agr activity compared to UAMS-1. Furthermore, *nos* promoter activity and RNA levels were significantly stronger in AH1263 relative to UAMS-1 during late-exponential low-oxygen growth, when *nos* expression is maximal. Global regulators Rex and MgrA were also implicated as negative regulators of low-oxygen *nos* promoter activity in UAMS-1. Collectively, these results provide new insight into factors that control *nos* expression.

**IMPORTANCE:**

Bacterial nitric oxide synthase (bNOS) has recently emerged in several species as a key player in resistance to stresses commonly encountered during infection. Although *Staphylococcus aureus* (sa)NOS has been suggested to be a promising drug target in *S. aureus*, an obstacle to this in practice is the existence of mammalian NOS, whose oxygenase domain is like bacterial NOS. Increased understanding of the *nos* regulatory network in *S. aureus* could allow targeting of saNOS through its regulators, bypassing the issue of also inhibiting mammalian NOS. Furthermore, the observed strain-dependent differences in *S. aureus nos* regulation presented in this study reinforce the importance of studying bacterial NOS regulation and function at both the strain and species levels.

## INTRODUCTION

*Staphylococcus aureus* is a highly virulent, opportunistic pathogen with diverse clinical presentations, capable of infecting almost every organ in the human body. The emergence of antibiotic-resistant strains of *S. aureus* such as methicillin-resistant *S. aureus* (MRSA) has created a widespread issue in clinical medicine, with some isolates resistant to nearly all commonly used antibiotics. The Centers for Disease Control and Prevention reports approximately 300,000 hospitalizations from *S. aureus* infection per year, and MRSA is recognized as a leading cause of hospital-acquired infections (1). A primary contributor to the success of *S. aureus* as a pathogen is its flexible and adaptable metabolism and virulence, as this bacterium is capable of fluctuating between multiple forms of respiration and fermentation and implementing a wide variety of virulence machinery.

*S. aureus* nitric oxide synthase (saNOS) has recently emerged as a key player in aerobic respiration as well as the switch between aerobic and anaerobic metabolism (2 – 5). NOS produces nitric oxide (NO) via the two-step oxidation of L-arginine to L-citrulline and NO (6). While mammalian NOS contains a reductase domain that transfers electrons from NADPH to the oxygenase domain where this reaction occurs (7), bacterial NOS contains only the oxygenase domain and is thought to promiscuously use available reductase partners (8). In mammals, NO is an important cellular signaling molecule involved in nervous system signaling (9, 10), regulating blood pressure (11, 12), and mitochondrial respiration (13, 14), as well as the immune response against pathogens as a cytotoxic agent (15). Bacterial NOS is found in some Gram-positive genera such as *Staphylococcus*, *Bacillus*, *Streptomyces*, and *Deinococcus*, with certain functions of this enzyme appearing to differ by genus (16). *S. aureus* NOS has been implicated in oxidative stress resistance (17 – 19), antibiotic tolerance (19, 20), virulence (18, 19, 21), heme stress resistance (22), the switch to nitrate-based respiration during low-oxygen growth (5), and aerobic respiration (2 – 4). An *S. aureus* UAMS-1 *nos* mutant demonstrated altered aerobic respiration, including increased membrane potential, increased respiratory dehydrogenase activity, and increased accumulation of reactive oxygen species (ROS) (2). This points to a role for saNOS in the modulation of aerobic respiration, although the exact mechanism has yet to be elucidated.

Despite the emerging appreciation for the importance of saNOS in *S. aureus* physiology and virulence, surprisingly little is understood about how the expression of *nos* is regulated. Expression of *nos* is upregulated under low-oxygen growth relative to aerobic growth (18), and the two-component regulatory system SrrAB has been identified as a positive regulator of *nos* responsible for increased *nos* expression during low-oxygen growth (3, 23). SrrAB is thought to sense and respond to the reduced state of the respiratory menaquinone pool to regulate genes in response to low oxygen and nitrosative stress (24, 25). Under nitrosative stress, SrrAB upregulates certain genes involved in the nitrosative stress response, anaerobic metabolism, and cytochrome biosynthesis (25) which have also been shown by transcriptome studies to be upregulated in the *nos* mutant under aerobic respiratory conditions (2). In addition, the interplay between saNOS and SrrAB appears to be more complicated than one-way regulation, as an *S. aureus nos srrAB* mutant displays distinct changes in physiology and gene expression relative to wild-type and single-mutant strains (3). The global transcriptional regulator MgrA was also recently shown to affect *nos* expression under anaerobic and nitrosative stress conditions (26). In this study, we further characterize SrrAB and MgrA as regulators of *nos*, determine the effect of other candidate *nos* regulators and strain dependency on *nos* expression, and identify the transcriptional start site (TSS) of the *S. aureus nos* transcript.

## MATERIALS AND METHODS

### Bacterial strains and culture conditions

The bacterial strains and plasmids used in this study are listed in Table S1. All experiments were performed with strains UAMS-1 (wild type, USA200 strain), KB6004 (UAMS-1 Δ*srrAB* mutant), KR1300 (UAMS-1 Δ*agr* mutant), KR6300 (UAMS-1 Δ*srrAB* Δ*agr* mutant), UAMS-1 *rex::kan* mutant, UAMS-1 Δ*mgrA*, AH1263 (wild type, USA300 strain), and JLB316 (AH1263 Δ*agr* mutant). These strains each containing plasmid pJB185 (27, 28), pJBnos1, or pJBnos2 were used in β-galactosidase assays. For each experiment, strains were freshly streaked from −80°C glycerol stocks and grown at 37°C on tryptic soy agar (TSA) with the following selective antibiotics, as appropriate, at the indicated concentrations: kanamycin (Kan) at 50 µg/mL, erythromycin (Erm) at 2  µg/mL, and chloramphenicol (Cm) at 5  µg/mL. Individual colonies were then subcultured for approximately 15 h in tryptic soy broth containing 14 mM glucose (TSB + G; with antibiotic as appropriate) at 37°C with shaking at 250 rpm. For aerobic and low-oxygen growth experiments, cultures were inoculated from overnight cultures to a final optical density (OD) at 600 nm (OD_600_) of 0.025. For aerobic growth conditions, cultures were grown at 37°C in 40 mL of TSB + G in 500 mL Erlenmeyer flasks (1:12.5 vol/flask ratio) and shaken at 250 rpm. For low-oxygen growth conditions, cultures were grown at 37°C in 350  mL of TSB + G in 500 mL Erlenmeyer flasks (1:1.4 vol/flask ratio) without shaking.

### Generation of *S. aureus* mutants

For the generation of UAMS-1 *agr* and *srrAB agr* mutants, primer sets agr_5′F + agr_5′R and agr_3′F + agr_3′R (Table S2) were used to PCR amplify two segments from UAMS-1 genomic DNA overlapping the 5′ and 3′ ends of the *agr* locus. The 5′ segment contained restriction sites EcoRI and XhoI on the 5′ and 3′ ends, respectively; the 3′ segment contained restriction sites XhoI and SalI on the 5′ and 3′ ends, respectively. Each segment was separately cloned into pCR-Blunt, sequenced, cut out by restriction digest, and ligated to each other and into the temperature-sensitive allele replacement vector pJB38 (29), which was digested with EcoRI and SalI. The ligation of the two segments eliminates *RNAII* (*agrBDCA*) as well as *RNAIII,* creating a complete *agr* deletion allele. Plasmid pJB38-Δ*agr* was then phage transduced from *S. aureus* RN4220 into UAMS-1 (wild type) or KB6004 (UAMS-1 Δ*srrAB*). Once its presence in the target strain was confirmed, a temperature-sensitive allele replacement event was initiated by growth at 43°C (the nonpermissive temperature for plasmid replication) on TSA plus 10 µg/mL Cm to promote chromosomal integration via homologous recombination at the *agr* locus, as described in (30). A second recombination event was induced by growing a single isolated colony in TSB (no antibiotic) for 5 days at 30°C with subculturing every 24 h. Screening for the loss of the plasmid was completed by picking and patching colonies onto TSA and TSA plus 5 µg/mL Cm. PCR with primers agr_up_F + agr_down_R (Table S2), located upstream and downstream of the *agr* locus, was used to confirm the presence of the *agr* deletion allele in strains UAMS-1 and KB6004.

For generation of JLB316 (AH1263 *agr* mutant), the regions upstream of RNAIII and downstream of *agrA* were amplified by PCR using primers listed in Table S2 and cloned into pJB38 (29). Briefly, the downstream fragment was amplified from the AH1263 genome using primers NS24 and JBKU123, digested with KpnI and SalI, and ligated into the same sites of pJB38 to generate pMP8. Next, the upstream fragment was amplified using primers JBKU120 and JBKU121, digested with EcoRI and NheI and cloned into the same sites of pMP8 to create pMP9, which was subsequently moved into RN4220 and AH1263 by electroporation and phage transduction, respectively. Allele replacement mutagenesis was then performed as described in references (29, 30).

For generation of the *rex* mutant, the previously created pJF102 plasmid containing Δ*rex::kan* was used (31). This plasmid was phage transduced into wild-type UAMS-1, with growth at 30°C for all steps. Once its presence in the target strain was confirmed, a temperature-sensitive allele replacement event was initiated by growth at 43°C (nonpermissive temperature for plasmid replication) on TSA plus 50 µg/mL Kan to promote the integration of the plasmid into the chromosome via homologous recombination at the *rex* locus. A second recombination event was induced by growing a single isolated colony in TSB (no antibiotic) for 5 days at 30°C with subculturing every 24 h. Screening for the loss of the vector was completed by picking and patching colonies onto TSA plus 50 µg/mL Kan and TSA plus 5 µg/mL Cm. PCR was used to confirm the presence of the *rex::kan* insertion allele.

### *nos-lacZ* reporter design

**Fig 1:**
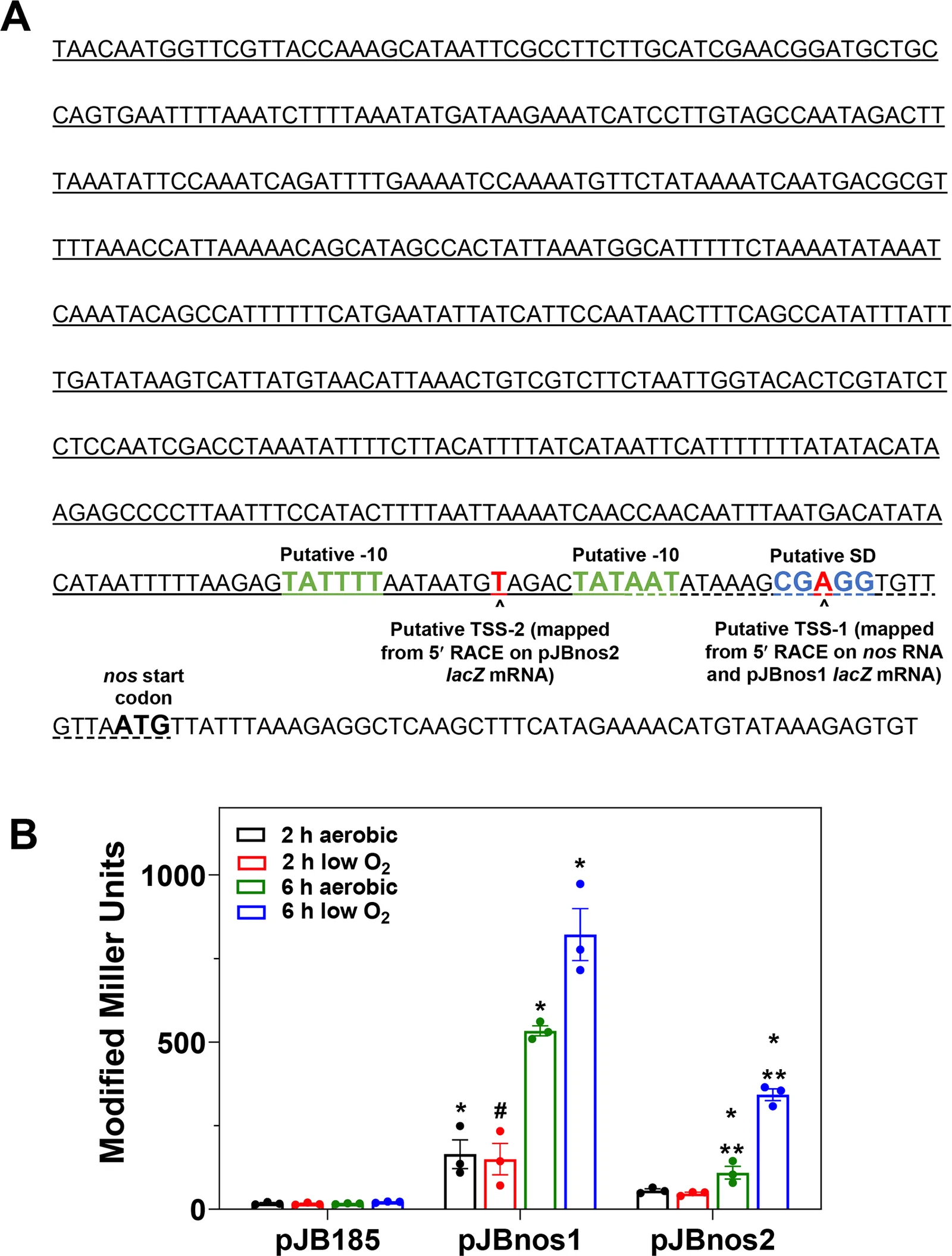
Diagram of the *nos* promoter region (**A**) and validation of growth-phase- and oxygen-dependent pJBnos1 and pJBnos2 promoter activities (**B**). (A) The genome-annotated start codon (black bolded) and upstream putative Shine-Dalgarno sequence (SD; blue bolded) are indicated. The transcriptional start site (TSS-1) identified by 5′ rapid amplification of cDNA ends (RACE) using *nos* cDNA template from UAMS-1 and AH1263, and *lacZ* cDNA template from UAMS-1 pJBnos1 was mapped to an adenine nucleotide (red bold) located in the predicted SD sequence. 5′ RACE using *lacZ* cDNA from UAMS-1 pJBnos2 mapped a second TSS to a thymine nucleotide (red bold; TSS-2) located 30 bp upstream to the *nos* ATG start codon. Putative −10 elements (green bold) are also indicated upstream of each TSS. The region cloned upstream of *lacZ* in pJBnos1 is indicated by both the solid underline and dotted underline, while the region cloned upstream of *lacZ* in pJBnos2 contains only the solid underlined sequence. (**B**) β-galactosidase activity of pJB185 (promoterless), pJBnos1, and pJBnos2 in wild-type *S. aureus* UAMS-1 was assessed in cell pellets harvested from aerobic or low-oxygen TSB + G cultures at 2- and 6-h growth, as described in Materials and Methods. Data are reported in modified Miller units. All data represent the average from three independent experiments; error bars indicate the standard error of the mean (SEM). *P < 0.05 (Holm-Sidak method) compared to pJB185 at each time point and growth condition; **P < 0.05 (Holm-Sidak method) compared to pJBnos1 at each time point and growth condition; ^#^ *P* < 0.05 (Tukey’s test) compared to pJB185 at each time point and growth condition.

Plasmid pJB185 (27) containing a codon-optimized *lacZ* gene was used to create *nos-lacZ* fusion reporter plasmids pJBnos1 and pJBnos2. The primers used to amplify the *nos* promoter region segments (approximately 500 bp region upstream of the *nos* ATG start codon) cloned into each reporter construct (nos_F + nos_R for pJBnos1 and nos2_F + nos2_R for pJBnos2) are listed in Table S2. The regions of the DNA sequence upstream to *nos* cloned in each reporter plasmid are also indicated in Fig. 1A. The region cloned upstream of *lacZ* in pJBnos1 includes the *nos* predicted start codon and a 508 bp region upstream, including the putative Shine-Dalgarno (SD) sequence. Plasmid pJBnos2 contains the 500 bp region upstream to the predicted SD sequence and does not include this or the *nos* start codon. Instead, a non-native SD sequence and translation enhancer region (28) present upstream of *lacZ* in pJB185 are used for translation in this construct. Briefly, the *nos* promoter regions were each PCR amplified from UAMS-1 genomic DNA, cloned into pCR-Blunt, sequenced, cut out by restriction digest, and ligated into pJB185 upstream of the codon-optimized *lacZ* gene. Restriction enzymes (EcoRI and XbaI for pJBnos1; EcoRI and BamHI for pJBnos2) were chosen to include or eliminate the appropriate portions of the pJB185 sequence. Plasmid constructs were phage transduced from RN4220 into UAMS-1, KB6004, KR1300, KR6300, *rex, mgrA,* AH1263, and JLB316, as indicated in each experiment.

### Mapping the *nos* TSS

A 5′ rapid amplification of cDNA ends (RACE) was performed according to a protocol provided by Life Technologies. Briefly, total RNA was isolated from *S. aureus* UAMS-1 and AH1263 cultures grown for 6 h (late exponential growth phase) in low-oxygen conditions using the RNeasy Mini Kit (Qiagen) as previously described (32). *nos* cDNA was then generated using the iScript Select cDNA Synthesis Kit (Bio-Rad) and the primer nos-GSP1 (Table S2). cDNA was purified using the Zymo Clean and Concentrator kit (Zymo Research) and used in a homopolymeric tailing reaction with 2 mM dCTP and 15 units of TdT (Invitrogen). The reaction product was amplified further in several rounds of PCR using nested gene-specific primers (nos-GSP2 and nos-GSP3; Table S2), and products were visualized on a 1% agarose gel using gel electrophoresis. Once a suitable amount of DNA was visualized, products were sent for Sanger sequencing (Genewiz) with primers nos-GSP3 and nos-screen (Table S2). The TSS (or the first nucleotide of the mRNA) was then determined from sequencing results by locating the homopolymeric tail and the first nucleotide following it. Similar methods were used with *lacZ*-specific primers (lacZ-GSP1, lacZ-GSP2, lacZ-GSP3, and lacZ-screen; Table S2) to map the TSS in *nos-lacZ* fusion plasmids pJBnos1 and pJBnos2. Sanger sequencing results are provided in Supplementary file 1.

### β-galactosidase assays (modified Miller assays)

All *S. aureus* wild-type and mutant strains containing pJBnos1, pJBnos2, or pJB185 (promoterless vector control) were grown in aerobic and/or low-oxygen conditions as described above. Cell pellets were harvested at 2-h (early exponential phase) and/or 6-h growth (late exponential phase) in each condition, with OD_600_ recorded at each time point. At 2 h, cells were harvested from a 20 mL volume of aerobic cultures and a 40 mL volume of low-oxygen cultures by centrifugation at 4,500 rpm. At 6 h, 5 mL was harvested from aerobic cultures and 20 mL from low-oxygen cultures by centrifugation at 4,500 rpm. Pellets were stored at −80°C until further processing. For isolation of cellular protein, cell pellets were thawed and resuspended in 800 µL 1× phosphate-buffered saline (PBS), then transferred to lysing matrix B tubes (MP Biomedicals) and lysed using a FastPrep (MP Biomedicals). The FastPrep was run at 6 m/sec for 30 s two times, with tubes placed in ice for 2 min between runs. Lysates were centrifuged at 4°C for 10 min at 12,000× *g*, then transferred and split into two new sterile tubes for use in BCA and β-galactosidase assays. The Pierce BCA protein assay kit (Thermo Fisher Scientific) was used to measure lysate protein concentration. β-galactosidase assays (modified Miller assays) were performed using ONPG substrate as described in reference (28). Activity was reported in modified Miller units (MU) with the formula MU = 1,000 × [*A*_420_/(*t* × *v* × mg/mL)], whereby *t* = time of reaction in minutes, *v* = volume (mL) of cell lysate, and mg/mL = protein concentration.

### RNA isolation and qPCR

To measure late-exponential-phase (6-h growth) *nos* gene expression in wild-type UAMS-1 and isogenic *srrAB*, *agr*, and *srrAB agr* mutant strains, as well as in wild-type AH1263 and isogenic *agr* mutant strain, aerobic and low-oxygen cultures of each strain were grown as described above. At 6-h growth, 5 mL of each aerobic culture and 20 mL of each low-oxygen culture were centrifuged at 4°C, 4,500 rpm for 10 min, and cell pellets were resuspended in RNAlater. Pellets were stored at −80°C until further processing. RNA was extracted using a FastPrep system with Lysing Matrix B tubes as previously described (32). RNA was further treated with Turbo DNase (Ambion Turbo DNA-free kit) to remove any contaminating DNA, and the RNA concentration and purity were quantified using a BioTek Take3 plate. Similar growth conditions and RNA isolation methods were used with strains UAMS-1, AH1263, UAMS-1 pJBnos1, and UAMS-1 pJBnos2 for 5′ RACE. For quantitative real-time PCR (qPCR), cDNA was synthesized from 0.75 µg of purified RNA using an iScript reverse transcriptase kit (Bio-Rad). Expression of *nos* was measured using iQ SYBR Green Supermix (Bio-Rad) and a CFX Connect real-time system (Bio-Rad). Relative fold expression normalized to that of the reference housekeeping gene *sigA* was calculated using the Livak method (2−^ΔΔCT^), as previously described (18, 33). The primers used for qPCR are listed in Table S2. For UAMS-1 and isogenic mutants, qPCR was performed on three biological samples with three technical replicates per sample. For AH1263, qPCR was performed on two biological samples with three technical replicates per sample.

### Statistical analysis

Unless otherwise indicated, assays were conducted in at least three independent experiments. Statistical analysis for all data was completed with SigmaPlot software (version 14; Systat Software Inc.) or GraphPad Prism (version 9; GraphPad Software, Dotmatics). Data were tested for normality and equal variance prior to choosing the appropriate parametric or nonparametric test.

## RESULTS AND DISCUSSION

### Validation of pJBnos1 as a reporter plasmid for *nos* expression

Plasmid pJBnos1, a *nos-lacZ* reporter plasmid containing the *nos* predicted SD sequence and start codon (Fig. 1A), was used to assess *nos* promoter activity in UAMS-1 during aerobic and low-oxygen growth (Fig. 1B), with parallel measurements of UAMS-1 containing promoterless pJB185 as a negative control. These experiments revealed that β-galactosidase activity in UAMS-1 pJBnos1 was significantly higher (*P* < 0.05) than the corresponding promoterless control strain at both time points and growth conditions. Furthermore, peak *nos* promoter activity in pJBnos1 occurred in low-oxygen culture conditions during late exponential phase (6 h), which correlates with the previously observed pattern of *nos* RNA levels being maximally expressed at late exponential phase during low-oxygen growth (18) (Fig. 1B). Taken together, these results suggest that *nos* promoter-driven β-galactosidase activity in pJBnos1 serves as a useful proxy for measuring *nos* expression.

### Annotation of the *nos* promoter

A 5′ RACE experiment was performed to identify the TSS of *nos*. In three independently performed and sequenced RACE reactions using RNA isolated from UAMS-1 6-h low-oxygen cultures [growth condition previously reported to have maximal *nos* transcript levels (18)], the TSS consistently mapped to an adenine nucleotide positioned 11 nucleotides upstream of the start codon (TSS-1), in the middle of the predicted SD sequence (Fig. 1A). This experiment was repeated on RNA isolated from strain AH1263 grown under the same conditions and confirmed the same mapped TSS-1 site as in UAMS-1. To further confirm this unusual result, 5′ RACE was also performed on RNA from UAMS-1 harboring the pJBnos1 reporter plasmid using *lacZ-*specific primers to identify the TSS of *lacZ* transcription. In two independently performed and sequenced RACE reactions, the TSS of *lacZ* in this construct mapped to the same adenine nucleotide (TSS-1) as *nos* 5′ RACE performed on RNA from UAMS-1 and AH1263 (Fig. 1A). To determine whether TSS-1 is required for transcription, we created another *nos-lacZ* construct in plasmid pJB185, termed pJBnos2, which contains the same *nos* promoter region as pJBnos1*,* but ends just upstream of TSS-1, thereby also eliminating the *nos* predicted SD sequence and start codon. Instead, pJBnos2 uses a non-native SD sequence engineered upstream of *lacZ* to drive translation. We predicted that pJBnos2 would have little to no β-galactosidase activity since it lacks TSS-1. Using growth conditions identical to UAMS-1 pJBnos1, modified Miller assays were used to measure β-galactosidase activity in UAMS-1 pJBnos2 (Fig. 1B). Although β-galactosidase activity was significantly (*P* < 0.05) decreased in pJBnos2 relative to pJBnos1 at both time points and growth conditions, *nos* promoter activity at late exponential phase in pJBnos2 was still significantly higher (*P* < 0.05) than the corresponding promoterless pJB185 control culture (Fig. 1B). Therefore, late-exponential-phase transcription of *lacZ* can occur to some extent in pJBnos2 under both aerobic and low-oxygen growth conditions, even in the absence of TSS-1.

Promoter activity of pJBnos2 followed a similar pattern to that observed with pJBnos1 (Fig. 1B) and previously quantified *nos* RNA levels (18), with peak activity observed in late-exponential-phase (6 h) low-oxygen cultures (Fig. 1B). 5′ RACE on the pJBnos2 construct using *lacZ*-specific primers, also performed in two independent reactions, identified a thymine nucleotide 30 bp upstream of the *nos* start codon (TSS-2) and 19 bp upstream of TSS-1 (Fig. 1A). It is unlikely that potential secondary structure in the TSS-1 region blocks readthrough of the reverse transcriptase reaction in the 5′ RACE assay, as repeating this experiment with an extended denaturation (10 min) and higher cDNA synthesis temperature (50°C) identified the same adenine TSS-1 site. Furthermore, the secondary structure in the TSS-1 region predicted by mFold modeling (34) (Fig. S1), combined with the predicted melting temperature of this structure (53.2°C), does not support the idea of a secondary structure interfering with the reverse transcriptase reaction. In addition, if this structure were formed, the reaction would presumably be blocked before the identified adenine nucleotide. Putative −10 promoter elements were estimated upstream of both TSS-1 and TSS-2 (Fig. 1A). It is possible TSS-2 could represent a less preferred TSS that is strong enough to initiate transcription in the absence of TSS-1, which may explain the decreased *nos* promoter activity of pJBnos2. Given the unusual location of TSS-1 (within the putative SD sequence), it possibly represents a site subject to posttranscriptional mRNA processing, such as by RNase III, RNase Y, RNase J1/J2, or another staphylococcal RNase. RNase III, which contributes to virulence gene regulation through degradation of the mRNA duplex that forms between Agr effector RNAIII and Protein A (*spa*) (35), is a double-strand specific RNase (36). As mRNA modeling consistently showed the region around TSS-1 as a single-stranded loop (Fig. S1), this RNase would likely only cleave at this position if there were an interaction with another RNA. RNase Y, on the other hand, cleaves single-stranded A-rich or AU-rich sequences (37 – 39); the site of TSS-1 does not seem to meet this criterion, as it is surrounded by the CGAGG of the putative SD sequence. RNase J1 and J2 form a heterodimer (RNase J) involved in the maturation and processing of 16S rRNA among many others (40 – 42). This complex has both endonuclease and 5′ to 3′ exonuclease activity, which is single-strand specific (39, 43, 44). While further investigation is required to determine whether mRNA processing indeed occurs at TSS-1, it is noteworthy that the *nos* transcript has not been specifically identified in published studies as being a candidate for processing and/or regulation in *S. aureus* by RNase III (45, 46) or RNase Y (37). However, a previously published exact mapping of transcriptome ends (EMOTE) assay identified a 5′ mono-phosphorylated guanine nucleotide (located immediately upstream of the TSS-1 adenine of *nos*) as being enriched in *S. aureus* RNase J1 and J1/J2 mutants, suggesting that this RNase complex may regulate the stability of the *nos* transcript (40).

Alternatively, TSS-1 may be an actual initiation site of transcription, and its close location to the ATG start codon may implicate the *nos* transcript as potentially having a leaderless organization with no identifiable SD sequence, a phenomenon not well characterized in *S. aureus*. Leaderless mRNAs permit translation without an identifiable SD sequence (47). It remains unclear exactly how translation is initiated from these leaderless transcripts, but several necessary elements have been implicated, including a local absence of secondary structure and simply the 5′ AUG of the mRNA (48, 49).

### Confirmation of SrrAB and MgrA as *nos* regulators in UAMS-1

**Fig 2:**
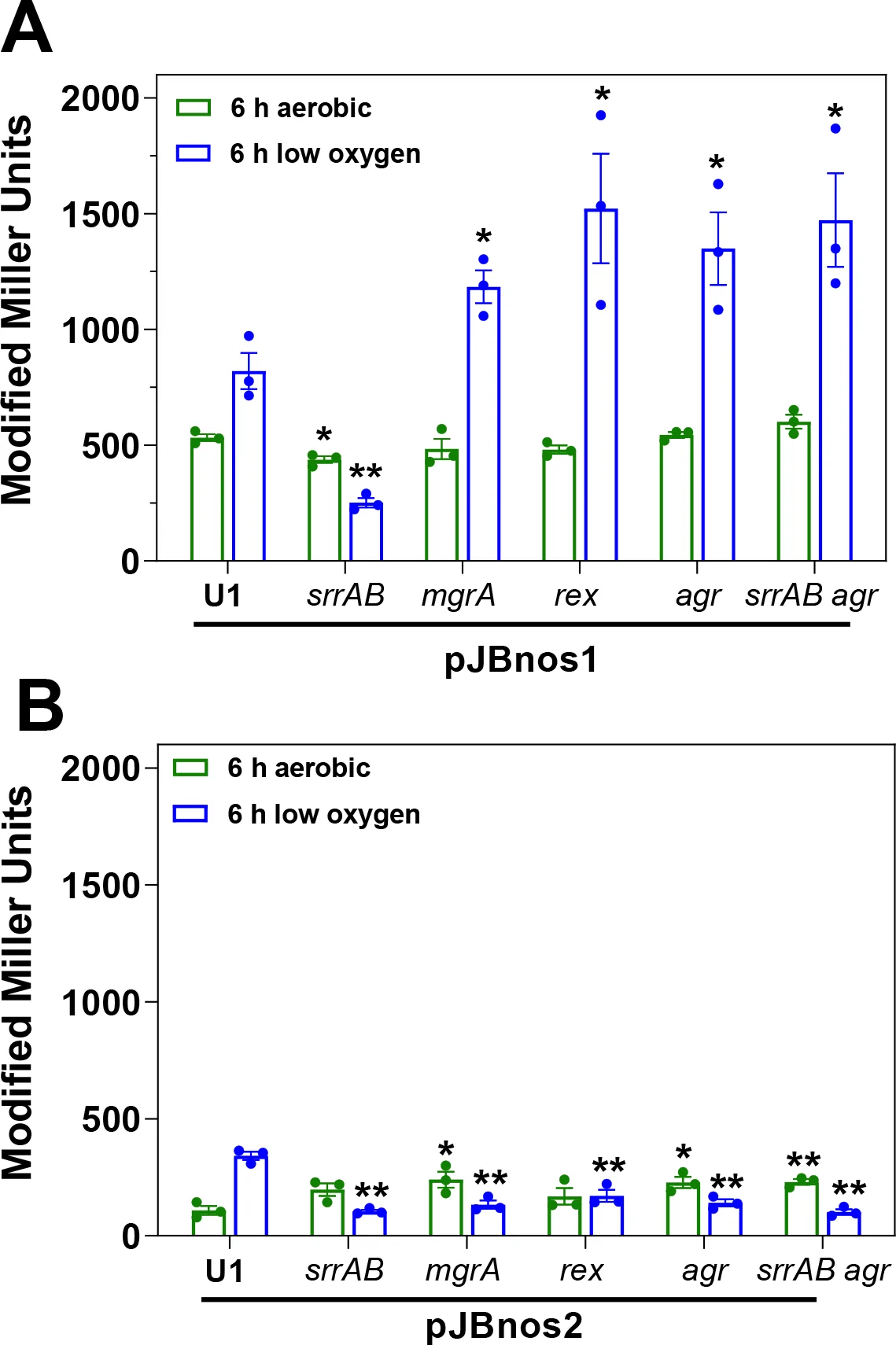
SrrAB, MgrA, Rex, and Agr affect *nos* expression in *S. aureus* UAMS-1. Cell pellets were harvested from aerobic or low-oxygen TSB + G cultures at 6 h for β-galactosidase assays, as described in Materials and Methods. The activity of pJBnos1 (**A**) and pJBnos2 (**B**) in wild-type UAMS-1 and isogenic *srrAB*, *mgrA*, *rex*, *agr*, and *srrAB agr* mutants is reported in modified Miller units. All data represent the average from three independent experiments; error bars indicate the standard error of the mean (SEM). *P < 0.05 (*t*-test) compared to UAMS-1 under the same growth condition, **P < 0.01 (*t*-test) compared to UAMS-1 under the same growth condition.

Understanding the *nos* regulatory network is an important piece of a more complete appreciation of *S. aureus* physiology, stress resistance, and virulence. The SrrAB TCS responds to both oxidative and nitrosative stress via sensing of the respiratory menaquinone pool and has both positive and negative effects on the expression of genes associated with metabolism (23), nitrosative stress (25), virulence (50), and biofilm formation (51, 52). In addition, SrrAB represses the expression of the Accessory Gene Regulator (Agr) TCS (50, 53), an important global quorum-sensing regulator [reviewed in references (54, 55)]. SrrAB has previously been identified as a positive regulator of *nos* mRNA levels, with *nos* RNA levels displaying a fourfold decrease by qPCR in a UAMS-1 *srrAB* mutant (3), and a sevenfold decrease by RNA microarray in a JE2 (LAC-derived strain) *srrA* mutant (23). The pattern of *nos* promoter-driven β-galactosidase activity in late-exponential-phase *srrAB* mutant pJBnos1 cultures (Fig. 2A) correlated with these previously published RNA results, whereby pJBnos1 promoter activity (containing TSS-1) in the *srrAB* mutant low-oxygen culture displayed a 3.3-fold significant decrease (*P* < 0.01) compared to UAMS-1. When these experiments were repeated with wild-type and *srrAB* mutant strains harboring pJBnos2 (lacking TSS-1) (Fig. 2B), similar expression trends were observed, albeit at overall lower activity levels relative to pJBnos1.

Global transcriptional regulator MgrA, a member of the MarR/SarA family that regulates a variety of genes related to cell aggregation and virulence (56 – 61), has also previously been implicated in *nos* regulation, with *nos* expression patterns in an *mgrA* mutant suggesting a repressive function of MgrA on *nos* expression during anaerobic growth, and a possible activating function under nitrosative stress conditions (26). β-galactosidase activity in late-exponential-phase *mgrA* pJBnos1 cultures confirmed that pJBnos1 promoter activity was significantly (*P* < 0.01) increased in the *mgrA* mutant during low-oxygen growth compared to UAMS-1 (Fig. 2A). However, different expression patterns were observed when these experiments were repeated with wild-type and *mgrA* mutant strains harboring pJBnos2 (Fig. 2B), whereby *nos* promoter activity was significantly decreased (*P* < 0.01) in the *mgrA* mutant during late-exponential-phase low-oxygen growth relative to wild type. This differential regulation of TSS-1 and TSS-2 driven *nos* promoter activity could be related to the observation that Cys12 (located in the dimerization domain) of MgrA is a target of both oxidation (62) and nitrosylation (63, 64). Oxidation of MgrA Cys12 was shown to weaken target promoter binding by MgrA (62), while nitrosylation of this amino acid strengthened promoter binding by MgrA (63). Interestingly, saNOS-derived NO was shown to be required for endogenous nitrosylation of MgrA (63), and *mgrA* expression was increased in a *nos* mutant (2), further reinforcing the regulatory interplay between saNOS and MgrA.

### Global regulators Rex and Agr affect *nos* expression

The redox-sensing transcriptional regulator Rex, a major player in *S. aureus* anaerobic gene regulation, is a known direct repressor of *srrAB* expression (65). Although inspection of the *nos* promoter region did not yield a convincing candidate Rex binding site, we pursued Rex as potentially involved in *nos* regulation due to its known connection to SrrAB and regulation of metabolic genes (65, 66). As mentioned above, SrrAB directly represses the expression of the Agr quorum-sensing system (50). It has not been confirmed whether SrrAB regulation of *nos* is directly mediated by SrrA promoter binding; therefore, SrrAB could also regulate *nos* transcript levels indirectly through another regulator, such as Agr. Evidence for a regulatory relationship between SrrAB, Agr, and saNOS can be found in previously published transcriptomic data of a UAMS-1 *nos* mutant (2), which revealed that expression of *srrAB* in the *nos* mutant was downregulated approximately 1.6-fold and *agr* operon genes were upregulated 1.7–2.3-fold. Similar Miller assays were performed using UAMS-1 and isogenic *rex* and *agr* mutants all harboring either the pJBnos1 or pJBnos2 reporter plasmid (Fig. 2). In the *rex* mutant, a 1.5–2-fold increase in β-galactosidase activity of pJBnos1 in late-exponential low-oxygen growth was observed (Fig. 2A). Rex is traditionally a repressor whose target genes are de-repressed in response to altered redox as oxygen levels decrease, so Rex-dependent *nos* repression could be a result of Rex binding directly to the *nos* promoter or indirectly acting through its role as a repressor of SrrAB. A recently published computational identification of *S. aureus* genome-wide Rex-binding sites using the DNA consensus sequence TTGTGAW6TCACAA (66) did not identify *nos* as a Rex-regulated gene, which corroborated the lack of a strong Rex-binding consensus sequence in our manual inspection of the *nos* promoter region. However, an indirect role for Rex may be involved in *nos* regulation, as transcript levels of several known Rex-regulated genes (*pflAB*, *ldh1*, *nar,* and *nir* operons) were also altered in a UAMS-1 *nos* mutant (2, 3), and decreased NADH levels were observed in late-exponential-phase *nos* mutant cells (2). β-galactosidase activity in late-exponential-phase UAMS-1 *agr* pJBnos1 cultures was also increased almost twofold during low-oxygen growth (Fig. 2A), suggesting a potential role for Agr as a negative regulator of *nos* expression under this growth condition. Interestingly, promoter activity in pJBnos2 was significantly (*P* < 0.01) decreased in both UAMS-1 *rex* (2-fold) and *agr* (2.4-fold) mutants under low oxygen growth (Fig. 2B), an opposite pattern to that observed in pJBnos1. Rex, Agr, and MgrA did not have a significant effect on aerobic regulation of *nos* expression in pJBnos1 (Fig. 2A), while pJBnos2 aerobic expression was modestly increased (*P* < 0.05) in *mgrA* and *agr* mutants (Fig. 2B). In addition, aerobic late-exponential-phase *nos* expression in pJBnos1 was modestly decreased (*P* < 0.05) in the *srrAB* mutant (Fig. 2A). Collectively, these data suggest that TSS-1 (in pJBnos1) and TSS-2 (in pJBnos2) may be subject to differential regulation by MgrA, Rex, and Agr in UAMS-1 when grown under low-oxygen conditions. It is unclear whether this could be related to direct regulation (e.g., altered affinity for the *nos* promoter by binding of one or more regulators which, in turn, drives expression from TSS-1 or TSS-2), or possibly indirect regulation of other DNA-binding proteins or RNase(s) acting on TSS-1.

### Agr and low-oxygen effects on *nos* expression are strain-dependent

**Fig 3:**
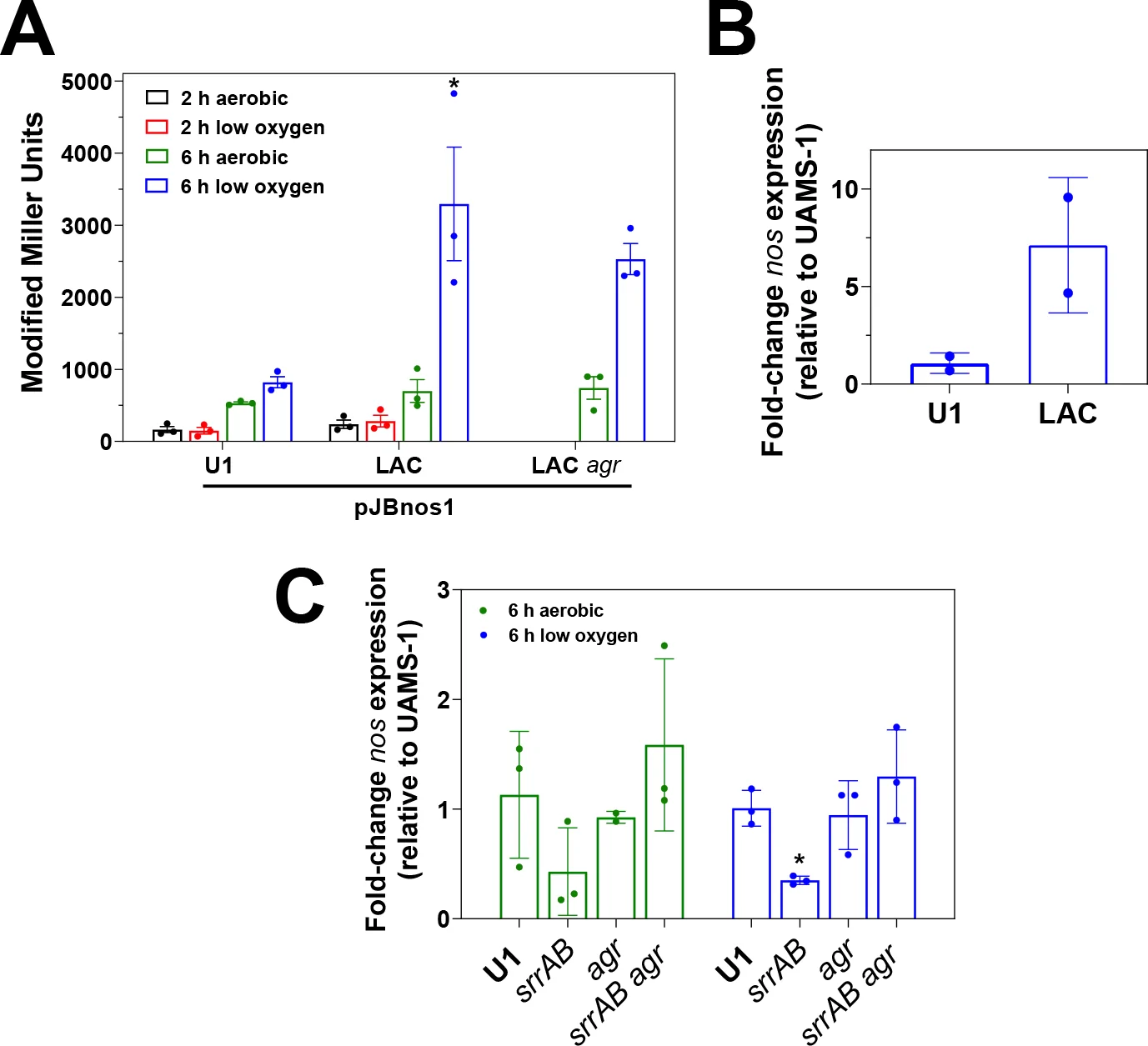
Low-oxygen and Agr-dependent *nos* expression is strain-dependent. (A) The activity of pJBnos1 in wild-type UAMS-1, AH1263 (LAC), and JLB316 (LAC *agr* mutant) reported in modified Miller units. Pellets were harvested from aerobic or low-oxygen TSB + G cultures at indicated time points for β-galactosidase assays, as described in Materials and Methods. All data represent the average from three independent experiments; error bars indicate the standard error of the mean (SEM). *P < 0.05 (two-tailed *t*-test) compared to UAMS-1 6-h low-oxygen time point. (**B)** *nos* RNA levels in UAMS-1 and AH1263 (LAC) were measured from low-oxygen TSB + G cultures of UAMS-1 and AH1263 at 6-h growth, as described in Materials and Methods. Quantitative real-time PCR was performed on reverse-transcribed cDNA from each sample using *nos*-specific primers. The Livak (2−^ΔΔCT^) method was used to determine the relative fold change of *nos* expression, using *sigA* expression as the reference gene and UAMS-1 as the calibrator. (**C)** *nos* RNA levels were measured in 6-h low-oxygen cultures of wild-type UAMS-1 and isogenic *srrAB, agr,* and *srrAB agr* mutants. Quantitative real-time PCR was performed on reverse-transcribed cDNA from each sample using *nos*-specific primers. The Livak (2−^ΔΔCT^) method was used to determine the relative fold change of *nos* expression, using *sigA* expression as the reference gene and the UAMS-1 sample as the calibrator for each growth condition. All data represent the average from two to three biological replicates; error bars indicate the SEM. *P < 0.05 (two-tailed *t*-test) compared to UAMS-1.

Agr activity in strain UAMS-1 (Agr class III) is relatively low compared to many other clinically relevant *S. aureus* strains (67, 68). To determine whether basal Agr activity impacts *nos* regulation, the pJBnos1 construct was moved into the USA300 LAC strain AH1263, which has much stronger Agr activity (Agr class I) (69 – 71), and JLB316 (isogenic AH1263 *agr* mutant). Surprisingly, *nos* promoter activity measured from pJBnos1 (containing the UAMS-1 *nos* promoter sequence) was significantly more active (~3.5-fold higher) in late-exponential-phase low-oxygen AH1263 cultures compared to UAMS-1 (Fig. 3A). This pattern of increased *nos* expression in AH1263 was confirmed by comparing late-exponential-phase low-oxygen *nos* RNA levels in both wild-type strains by qPCR (Fig. 3B). This striking difference in *nos* expression between UAMS-1 and AH1263 is not due to differences in *nos* promoter sequences, as BLAST comparison of the 522 bp upstream sequences to the *nos* ATG start codon from each strain revealed over 99% identity, with no nucleotide insertions, deletions, or substitutions in the 185 bp sequence immediately upstream to the ATG codon (data not shown). β-galactosidase activities measured during aerobic growth and early-exponential-phase low-oxygen growth in AH1263 also appeared to trend higher compared to UAMS-1, but these differences were not statistically significant (Fig. 3A). No significant differences in *nos* expression were observed between wild-type AH1263 and its isogenic *agr* mutant at any of the growth conditions and time points tested, indicating the involvement of Agr in *nos* regulation may be strain dependent and/or Agr class-dependent (Fig. 3A).

The striking difference in late-exponential-phase low-oxygen *nos* expression between UAMS-1 and AH1263 suggests that as-yet-unknown strain-specific differences in genetic regulators and/or physiology contribute to the control of *nos* expression. Although characterization of *S. aureus nos* mutants has revealed many conserved phenotypes across different strain backgrounds, including UAMS-1 and USA300 strains [increased pigmentation, increased oxygen consumption, increased tolerance to aminoglycoside antibiotics, decreased virulence (2 – 5, 18, 19)], there also appear to be some *nos* mutant metabolic phenotypes that are strain dependent. Aerobically grown UAMS-1 *nos* mutant cells display increased O_2_ consumption and increased respiratory dehydrogenase activity (2, 3, 5), supporting a role for saNOS in slowing aerobic respiration and minimizing endogenous ROS, possibly by NO competition with O_2_ at the heme active site of cytochromes (72 – 75). However, nitrite from saNOS-derived endogenous NO was postulated to promote aerobic respiration in *S. aureus* JE2 (a USA300 strain) by stimulating quinol oxidase activity, as the growth defect of this *nos* mutant could be rescued by nitrate or nitrite (but not urea or ammonia) and this rescue was abolished in a *nos qox* double mutant (4). In addition, *nos* mutants in the USA300 background appear to have a more pronounced aerobic growth defect (2, 4) compared to UAMS-1 (2, 3), which could relate to the increased basal expression of *nos* observed between these two strain backgrounds (Fig. 3).

### Agr is epistatic to SrrAB in regulating *nos* expression in *S. aureus* UAMS-1

A *srrAB agr* double mutant was made in strain UAMS-1 to investigate possible regulatory interaction between these two systems. β-galactosidase assays were performed in the double mutant with pJBnos1 (TSS-1) and pJBnos2 (TSS-2), and activity was compared to that in wild type and the respective single mutants (Fig. 2A and B). Interestingly, the activity of pJBnos1 in the *srrAB agr* double mutant phenocopied the *agr* single mutant. As previously observed, *nos* promoter activity was decreased in the *srrAB* mutant and increased in the *agr* mutant in late-exponential-phase low-oxygen cultures (Fig. 2A). The *srrAB agr* double mutant mimicked the *agr* single mutant, with the activity of pJBnos1 increased approximately twofold in low oxygen (Fig. 2A). Activity of pJBnos2 in the *agr nos* mutant also phenocopied the *agr* mutant, displaying reduced expression under low-oxygen growth and modestly increased expression under aerobic growth (Fig. 2B).

qPCR was also used to measure *nos* RNA levels in wild-type UAMS-1 and its isogenic *srrAB, agr,* and *srrAB agr* mutants. RNA was isolated from 6-h aerobic and low-oxygen cultures, the same growth conditions used in the β-galactosidase assays, and *nos* transcript levels were expressed as a fold change relative to the wild-type sample in each condition (Fig. 3C). Decreased *nos* RNA levels were observed in the *srrAB* mutant, which has been consistently observed here (Fig. 2 and 3C) and in previously published studies (3, 23). In the *agr* and *srrAB agr* mutants, significant differences in *nos* transcript levels compared to the wild type (Fig. 3C) were not observed. While this differs from the increase seen with pJBnos1 in β-galactosidase assays (Fig. 2A), the fact that the addition of an *agr* mutation reverses the decreased *nos* promoter activity and transcript levels of the *srrAB* mutant holds true.

Agr is likely an indirect and/or posttranscriptional negative regulator of *nos* expression, since (i) no putative AgrA-binding site was identified in the *nos* promoter region and (ii) most Agr regulation is posttranscriptional through the activity of RNAIII. Given that SrrAB is known to repress Agr (50), decreased *nos* expression in the *srrAB* mutant could be a consequence of de-repressed Agr activity, which, in turn, could repress *nos* expression. This scenario is consistent with increased low-oxygen pJBnos1 expression observed in the UAMS-1 *agr* mutant (Fig. 2A). However, data from our laboratory and others have shown what seems to be an extensive interplay between saNOS and SrrAB (3, 4). SrrAB upregulates several respiratory genes in response to altered respiration caused by a *nos* mutation (3, 4). In addition, saNOS and SrrAB were observed to be epistatic to each other in regard to different phenotypes; SrrAB is epistatic to saNOS in the expression of nitrosative stress genes, NAD^+^/NADH ratio, and *in vivo* virulence, while saNOS is epistatic to SrrAB in aminoglycoside tolerance (3). This, along with the decreased expression of *nos* in a *srrAB* mutant, would seem to support SrrAB as a direct positive regulator of *nos*. Although it does remain possible that saNOS and SrrAB are independently involved in similar respiration-related processes and that decreased *nos* expression in the *srrAB* mutant is attributed to a stronger repressive effect of Agr, another possible explanation is that SrrAB and Agr independently regulate *nos* expression, with SrrAB as a positive regulator and Agr as a negative regulator. Decreased *nos* expression in the *srrAB* mutant could be an additive effect from both regulators, with SrrAB not present to activate *nos* and increased *agr* expression caused by the *srrAB* mutation leading to further *nos* repression. In this scenario, the observation that *nos* expression in the UAMS-1 *srrAB agr* double mutant mimics increased *nos* expression in the *agr* single mutant could mean that mutation of *agr* induces a SrrAB-independent mechanism of *nos* activation, or that there is an alternative unknown regulator that is regulated or repressed by Agr, that is responsible for activating *nos* expression.

### Conclusions

**Fig 4:**
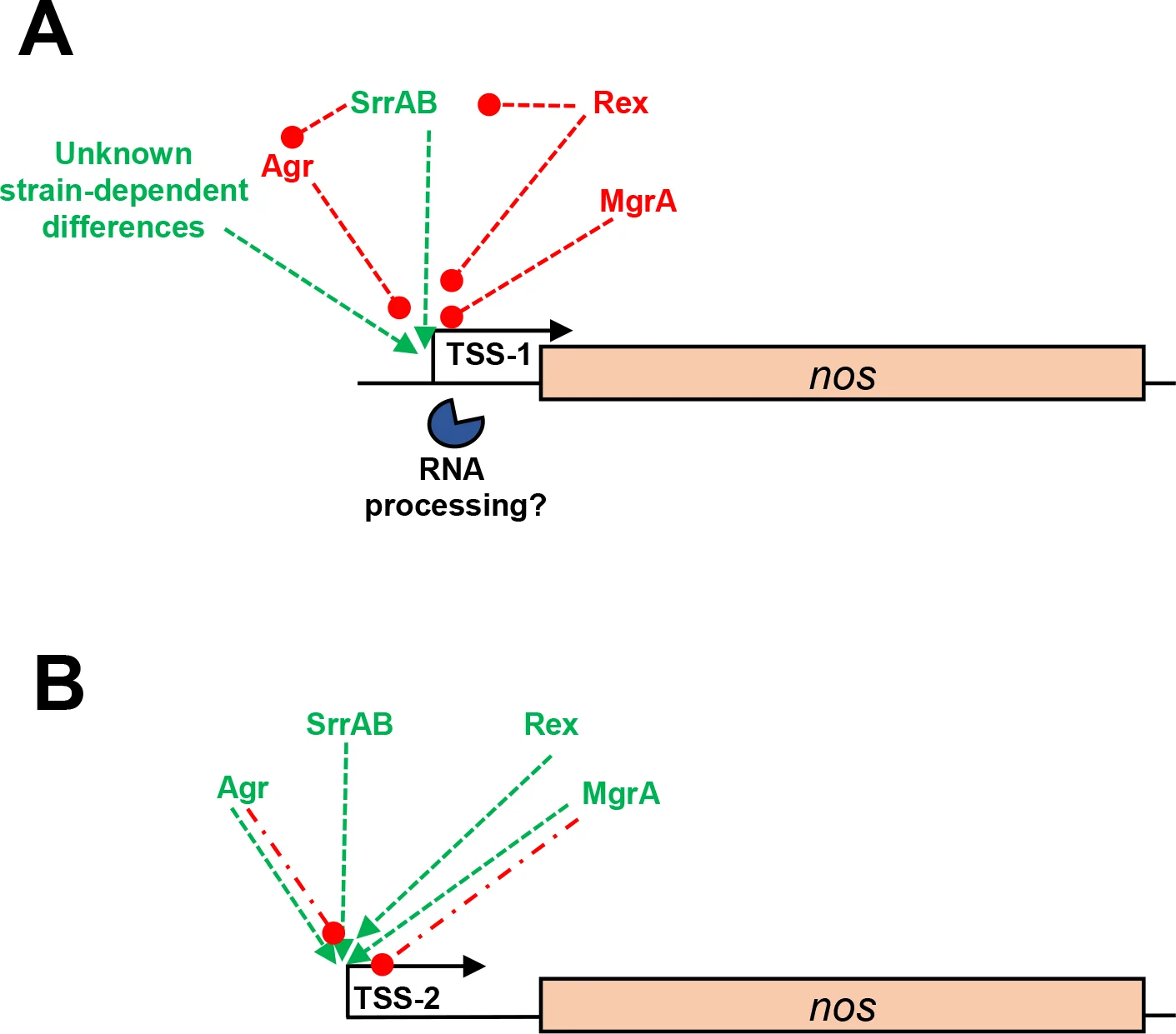
Summary of *nos* regulation. (A) Based on pJBnos1 data in this paper, SrrAB and unknown strain-dependent factors are positive regulators (green) of TSS-1-dependent *nos* expression during low-oxygen growth, whereas Agr, Rex, and MgrA appear to have repressive functions (red) on TSS-1-dependent *nos* expression in strain UAMS-1. TSS-1 may represent a site for RNA processing or possibly represents leaderless translation. (**B**) Based on pJBnos2 data, TSS-2-dependent *nos* expression is subject to positive regulation by SrrAB under both low-oxygen and aerobic growth conditions, whereas MgrA, Rex, and Agr-dependent regulation are dependent on the growth condition (all three regulators positively regulate TSS-2-dependent *nos* expression during low-oxygen growth, whereas Agr and MgrA repress expression under aerobic growth conditions).

Deciphering the *nos* regulatory network in *S. aureus* is key to further understanding how saNOS is involved in aerobic and anaerobic metabolism, stress resistance, and virulence. Overall, this study has identified two potential *nos* TSSs that may be differentially regulated by growth conditions and/or specific global regulatory systems, as summarized in Fig. 4. This study has also further confirmed the roles of SrrAB and MgrA in *nos* regulation and has identified Agr and Rex as putative negative regulators of *nos* expression in low-oxygen UAMS-1 cultures. Although the effects of potential interactions between these regulators, TSS-1, and TSS-2 on *nos* expression needs further study, it is possible that Agr acts through MgrA, as RNAIII has been shown to increase MgrA levels through stabilization of the mRNA (76). In addition, the increased low-oxygen *nos* expression observed in a *rex* mutant could be attributed to SrrAB, as Rex is a known repressor of *srrAB* expression (65). It is not likely a coincidence that all identified putative *nos* regulators are deeply intertwined with each other with respect to their roles in regulating metabolic genes; therefore, it is not surprising that mutation of multiple regulators could be causing similar effects on *nos* expression. We have also identified strain-dependent differences in *nos* expression and regulation, highlighting the importance of studying saNOS in different strain backgrounds. In addition, we have mapped two potential transcriptional start sites within the *nos* promoter, with TSS-1 appearing to be the stronger of the two. The location of TSS-1 within the putative SD sequence suggests that the *nos* transcript may be subject to unusual regulations such as RNA processing or leaderless translation. While further studies are needed to confirm the exact mechanisms behind this regulatory network, this current work contributes to our understanding of how *nos* expression is controlled, particularly in low-oxygen conditions, to function in the switch from aerobic to anaerobic metabolism (5), helping to build a more complete picture of how *nos* integrates into the transcriptional network that controls and responds to *S. aureus* metabolism.

## SUPPLEMENTAL MATERIAL

Supplemental Text S1 spectrum.01688-23-s0001.docx 5' RACE sequences.

Supplemental Figure S1 spectrum.01688-23-s0002.pdf Supplemental Figure S1.

Supplemental Tables S1 and S2 spectrum.01688-23-s0003.docx Supplemental Table S1 - strains and plasmids. Table S2 - primers.

ASM does not own the copyrights to Supplemental Material that may be linked to, or accessed through, an article. The authors have granted ASM a non-exclusive, world-wide license to publish the Supplemental Material files. Please contact the corresponding author directly for reuse.

## References

[1] Turner NA , Sharma-Kuinkel BK , Maskarinec SA , Eichenberger EM , Shah PP , Carugati M , Holland TL , Fowler VG . 2019. Methicillin-resistant Staphylococcus aureus: an overview of basic and clinical research. Nat Rev Microbiol 17:203–218. doi:10.1038/s41579-018-0147-4 30737488 PMC6939889

[2] Mogen AB , Carroll RK , James KL , Lima G , Silva D , Culver JA , Petucci C , Shaw LN , Rice KC . 2017. Staphylococcus aureus nitric oxide synthase (saNOS) modulates aerobic respiratory metabolism and cell physiology. Mol Microbiol 105:139–157. doi:10.1111/mmi.13693 28431199 PMC5641370

[3] James KL , Mogen AB , Brandwein JN , Orsini SS , Ridder MJ , Markiewicz MA , Bose JL , Rice KC . 2019. Interplay of nitric oxide synthase (NOS) and SrrAB in modulation of Staphylococcus aureus metabolism and virulence. Infect Immun 87:e00570-18. doi:10.1128/IAI.00570-18 PMC6346124 30420450

[4] Chaudhari SS , Kim M , Lei S , Razvi F , Alqarzaee AA , Hutfless EH , Powers R , Zimmerman MC , Fey PD , Thomas VC . 2017. Nitrite derived from endogenous bacterial nitric oxide synthase activity promotes aerobic respiration. mBio 8:e00887-17. doi:10.1128/mBio.00887-17 28765220 PMC5539425

[5] Kinkel TL , Ramos-Montañez S , Pando JM , Tadeo DV , Strom EN , Libby SJ , Fang FC . 2016. An essential role for bacterial nitric oxide synthase in Staphylococcus aureus electron transfer and colonization. Nat Microbiol 2:16224. doi:10.1038/nmicrobiol.2016.224 27892921 PMC5451252

[6] Alderton WK , Cooper CE , Knowles RG . 2001. Nitric oxide synthases: structure, function and inhibition. Biochem J 357:593–615. doi:10.1042/0264-6021:3570593 11463332 PMC1221991

[7] Stuehr DJ . 1999. Mammalian nitric oxide synthases. Biochim Biophys Acta 1411:217–230. doi:10.1016/s0005-2728(99)00016-x 10320659

[8] Gusarov I , Starodubtseva M , Wang Z-Q , McQuade L , Lippard SJ , Stuehr DJ , Nudler E . 2008. Bacterial nitric-oxide synthases operate without a dedicated redox partner. J Biol Chem 283:13140–13147. doi:10.1074/jbc.M710178200 18316370 PMC2442334

[9] O’Dell TJ , Hawkins RD , Kandel ER , Arancio O . 1991. Tests of the roles of two diffusible substances in long-term potentiation: evidence for nitric oxide as a possible early retrograde messenger. Proc Natl Acad Sci U S A 88:11285–11289. doi:10.1073/pnas.88.24.11285 1684863 PMC53119

[10] Schuman EM , Madison DV . 1991. A requirement for the intercellular messenger nitric oxide in long-term potentiation. Science 254:1503–1506. doi:10.1126/science.1720572 1720572

[11] Rapoport RM , Draznin MB , Murad F . 1983. Endothelium-dependent relaxation in rat aorta may be mediated through cyclic GMP-dependent protein phosphorylation. Nature 306:174–176. doi:10.1038/306174a0 6316142

[12] Förstermann U , Mülsch A , Böhme E , Busse R . 1986. Stimulation of soluble guanylate cyclase by an acetylcholine-induced endothelium-derived factor from rabbit and canine arteries. Circ Res 58:531–538. doi:10.1161/01.res.58.4.531 2870826

[13] Brown GC . 1995. Nitric oxide regulates mitochondrial respiration and cell functions by inhibiting cytochrome oxidase. FEBS Lett 369:136–139. doi:10.1016/0014-5793(95)00763-y 7649245

[14] Lipton SA . 2001. Nitric oxide and respiration. Nature 413:119–121. doi:10.1038/35093186 11557961

[15] Kröncke KD , Fehsel K , Kolb-Bachofen V . 1997. Nitric oxide: cytotoxicity versus cytoprotection--how, why, when, and where? Nitric Oxide 1:107–120. doi:10.1006/niox.1997.0118 9701050

[16] Sudhamsu J , Crane BR . 2009. Bacterial nitric oxide synthases: what are they good for? Trends Microbiol 17:212–218. doi:10.1016/j.tim.2009.02.003 19375324

[17] Gusarov I , Nudler E . 2005. NO-mediated cytoprotection: instant adaptation to oxidative stress in bacteria. Proc Natl Acad Sci U S A 102:13855–13860. doi:10.1073/pnas.0504307102 16172391 PMC1236549

[18] Sapp AM , Mogen AB , Almand EA , Rivera FE , Shaw LN , Richardson AR , Rice KC . 2014. Contribution of the nos-pdt operon to virulence phenotypes in methicillin-sensitive Staphylococcus aureus. PLoS One 9:e108868. doi:10.1371/journal.pone.0108868 25275514 PMC4183505

[19] van Sorge NM , Beasley FC , Gusarov I , Gonzalez DJ , von Köckritz-Blickwede M , Anik S , Borkowski AW , Dorrestein PC , Nudler E , Nizet V . 2013. Methicillin-resistance Staphylococcus aureus bacterial nitric oxide synthase affects antibiotic sensitivity and skin abscess development. J Biol Chem 288:6417–6426. doi:10.1074/jbc.M112.448738 23322784 PMC3585076

[20] Gusarov I , Shatalin K , Starodubtseva M , Nudler E . 2009. Endogenous nitric oxide protects bacteria against a wide spectrum of antibiotics. Science 325:1380–1384. doi:10.1126/science.1175439 19745150 PMC2929644

[21] Shatalin K , Gusarov I , Avetissova E , Shatalina Y , McQuade LE , Lippard SJ , Nudler E . 2008. Bacillus anthracis-derived nitric oxide is essential for pathogen virulence and survival in macrophages. Proc Natl Acad Sci U S A 105:1009–1013. doi:10.1073/pnas.0710950105 18215992 PMC2242674

[22] Surdel MC , Dutter BF , Sulikowski GA , Skaar EP . 2016. Bacterial nitric oxide synthase is required for the Staphylococcus aureus response to heme stress. ACS Infect Dis 2:572–578. doi:10.1021/acsinfecdis.6b00081 27626297 PMC6610873

[23] Wilde AD , Snyder DJ , Putnam NE , Valentino MD , Hammer ND , Lonergan ZR , Hinger SA , Aysanoa EE , Blanchard C , Dunman PM , Wasserman GA , Chen J , Shopsin B , Gilmore MS , Skaar EP , Cassat JE . 2015. Bacterial hypoxic responses revealed as critical determinants of the host-pathogen outcome by TnSeq analysis of Staphylococcus aureus invasive infection. PLoS Pathog 11:e1005341. doi:10.1371/journal.ppat.1005341 26684646 PMC4684308

[24] Richardson AR , Dunman PM , Fang FC . 2006. The nitrosative stress response of Staphylococcus aureus is required for resistance to innate immunity. Mol Microbiol 61:927–939. doi:10.1111/j.1365-2958.2006.05290.x 16859493

[25] Kinkel TL , Roux CM , Dunman PM , Fang FC . 2013. The Staphylococcus aureus SrrAB two-component system promotes resistance to nitrosative stress and hypoxia. mBio 4:e00696–13. doi:10.1128/mBio.00696-13 24222487 PMC3892780

[26] Favazzo LJ , Gill AL , Farnsworth CW , Mooney RA , Gill SR . 2019. The response of nor and nos contributes to Staphylococcus aureus virulence and metabolism. J Bacteriol 201:e00107–19. doi:10.1128/JB.00107-19 30782631 PMC6456865

[27] Krute CN , Rice KC , Bose JL . 2017. VfrB is a key activator of the Staphylococcus aureus saers two-component system. J Bacteriol 199:e00828-16. doi:10.1128/JB.00828-16 28031278 PMC5309915

[28] Krute CN , Seawell NA , Bose JL . 2021. Measuring staphylococcal promoter activities using a codon-optimized β-galactosidase reporter. Methods Mol Biol 2341:37–44. doi:10.1007/978-1-0716-1550-8_6 34264459 PMC8898383

[29] Bose JL , Fey PD , Bayles KW . 2013. Genetic tools to enhance the study of gene function and regulation in Staphylococcus aureus. Appl Environ Microbiol 79:2218–2224. doi:10.1128/AEM.00136-13 23354696 PMC3623228

[30] Lehman MK , Bose JL , Bayles KW . 2016. Allelic exchange. Methods Mol Biol 1373:89–96. doi:10.1007/7651_2014_187 25646609

[31] Crooke AK , Fuller JR , Obrist MW , Tomkovich SE , Vitko NP , Richardson AR , Msadek T . 2013. CcpA-independent glucose regulation of lactate dehydrogenase 1 in Staphylococcus aureus. PLoS One 8:e54293. doi:10.1371/journal.pone.0054293 23342123 PMC3544828

[32] Patton TG , Rice KC , Foster MK , Bayles KW . 2005. The Staphylococcus aureus cidC gene encodes a pyruvate oxidase that affects acetate metabolism and cell death in stationary phase. Mol Microbiol 56:1664–1674. doi:10.1111/j.1365-2958.2005.04653.x 15916614

[33] Lewis AM , Rice KC . 2016. Quantitative real-time PCR (qPCR) workflow for analyzing Staphylococcus aureus gene expression. Methods Mol Biol 1373:143–154. doi:10.1007/7651_2014_193 25646613

[34] Zuker M . 2003. Mfold web server for nucleic acid folding and hybridization prediction. Nucleic Acids Res 31:3406–3415. doi:10.1093/nar/gkg595 12824337 PMC169194

[35] Huntzinger E , Boisset S , Saveanu C , Benito Y , Geissmann T , Namane A , Lina G , Etienne J , Ehresmann B , Ehresmann C , Jacquier A , Vandenesch F , Romby P . 2005. Staphylococcus aureus RNAIII and the endoribonuclease III coordinately regulate spa gene expression. EMBO J 24:824–835. doi:10.1038/sj.emboj.7600572 15678100 PMC549626

[36] Chevalier C , Huntzinger E , Fechter P , Boisset S , Vandenesch F , Romby P , Geissmann T . 2008. Staphylococcus aureus endoribonuclease III purification and properties. Methods Enzymol 447:309–327. doi:10.1016/S0076-6879(08)02216-7 19161850

[37] Marincola G , Schäfer T , Behler J , Bernhardt J , Ohlsen K , Goerke C , Wolz C . 2012. RNase Y of Staphylococcus aureus and its role in the activation of virulence genes. Mol Microbiol 85:817–832. doi:10.1111/j.1365-2958.2012.08144.x 22780584

[38] Shahbabian K , Jamalli A , Zig L , Putzer H . 2009. RNase Y, a novel endoribonuclease, initiates riboswitch turnover in Bacillus subtilis. EMBO J 28:3523–3533. doi:10.1038/emboj.2009.283 19779461 PMC2782095

[39] Durand S , Condon C . 2018. RNases and helicases in Gram-positive bacteria. Microbiol Spectr 6. doi:10.1128/microbiolspec.RWR-0003-2017 PMC11633581 29651979

[40] Linder P , Lemeille S , Redder P . 2014. Transcriptome-wide analyses of 5'-ends in RNase J mutants of a Gram-positive pathogen reveal a role in RNA maturation, regulation and degradation. PLoS Genet 10:e1004207. doi:10.1371/journal.pgen.1004207 24586213 PMC3937233

[41] Britton RA , Wen T , Schaefer L , Pellegrini O , Uicker WC , Mathy N , Tobin C , Daou R , Szyk J , Condon C . 2007. Maturation of the 5' end of Bacillus subtilis 16S rRNA by the essential ribonuclease YkqC/RNase J1. Mol Microbiol 63:127–138. doi:10.1111/j.1365-2958.2006.05499.x 17229210

[42] Durand S , Braun F , Helfer A-C , Romby P , Condon C . 2017. sRNA-mediated activation of gene expression by inhibition of 5'-3' exonucleolytic mRNA degradation. Elife 6:e23602. doi:10.7554/eLife.23602 28436820 PMC5419742

[43] Hausmann S , Guimarães VA , Garcin D , Baumann N , Linder P , Redder P . 2017. Both exo- and endo-nucleolytic activities of RNase J1 from Staphylococcus aureus are manganese dependent and active on triphosphorylated 5'-ends. RNA Biol 14:1431–1443. doi:10.1080/15476286.2017.1300223 28277929 PMC5711453

[44] Mathy N , Hébert A , Mervelet P , Bénard L , Dorléans A , Li de la Sierra-Gallay I , Noirot P , Putzer H , Condon C . 2010. Bacillus subtilis ribonucleases J1 and J2 form a complex with altered enzyme behaviour. Mol Microbiol 75:489–498. doi:10.1111/j.1365-2958.2009.07004.x 20025672

[45] Lioliou E , Sharma CM , Altuvia Y , Caldelari I , Romilly C , Helfer A-C , Margalit H , Romby P . 2013. In vivo mapping of RNA-RNA interactions in Staphylococcus aureus using the endoribonuclease III. Methods 63:135–143. doi:10.1016/j.ymeth.2013.06.033 23851283

[46] Lioliou E , Sharma CM , Caldelari I , Helfer A-C , Fechter P , Vandenesch F , Vogel J , Romby P . 2012. Global regulatory functions of the Staphylococcus aureus endoribonuclease III in gene expression. PLoS Genet 8:e1002782. doi:10.1371/journal.pgen.1002782 22761586 PMC3386247

[47] Leiva LE , Katz A . 2022. Regulation of leaderless mRNA translation in bacteria. Microorganisms 10:723. doi:10.3390/microorganisms10040723 35456773 PMC9031893

[48] Scharff LB , Childs L , Walther D , Bock R . 2011. Local absence of secondary structure permits translation of mRNAs that lack ribosome-binding sites. PLoS Genet 7:e1002155. doi:10.1371/journal.pgen.1002155 21731509 PMC3121790

[49] Brock JE , Pourshahian S , Giliberti J , Limbach PA , Janssen GR . 2008. Ribosomes bind leaderless mRNA in Escherichia coli through recognition of their 5'-terminal AUG. RNA 14:2159–2169. doi:10.1261/rna.1089208 18755843 PMC2553737

[50] Pragman AA , Yarwood JM , Tripp TJ , Schlievert PM . 2004. Characterization of virulence factor regulation by SrrAB, a two-component system in Staphylococcus aureus. J Bacteriol 186:2430–2438. doi:10.1128/JB.186.8.2430-2438.2004 15060046 PMC412142

[51] Mashruwala AA , Guchte A van de , Boyd JM . 2017. Impaired respiration elicits SrrAB-dependent programmed cell lysis and biofilm formation in Staphylococcus aureus. Elife 6:e23845. doi:10.7554/eLife.23845 28221135 PMC5380435

[52] Ulrich M , Bastian M , Cramton SE , Ziegler K , Pragman AA , Bragonzi A , Memmi G , Wolz C , Schlievert PM , Cheung A , Döring G . 2007. The staphylococcal respiratory response regulator SrrAB induces ica gene transcription and polysaccharide intercellular adhesin expression, protecting Staphylococcus aureus from neutrophil killing under anaerobic growth conditions. Mol Microbiol 65:1276–1287. doi:10.1111/j.1365-2958.2007.05863.x 17697253

[53] Wilde AD , Snyder DJ , Putnam NE , Valentino MD , Hammer ND , Lonergan ZR , Hinger SA , Aysanoa EE , Blanchard C , Dunman PM , Wasserman GA , Chen J , Shopsin B , Gilmore MS , Skaar EP , Cassat JE . 2015. Bacterial hypoxic responses revealed as critical determinants of the host-pathogen outcome by TnSeq analysis of Staphylococcus aureus invasive infection. PLoS Pathog 11:e1005341. doi:10.1371/journal.ppat.1005341 26684646 PMC4684308

[54] Cheung GYC , Wang R , Khan BA , Sturdevant DE , Otto M . 2011. Role of the accessory gene regulator agr in community-associated methicillin-resistant Staphylococcus aureus pathogenesis. Infect Immun 79:1927–1935. doi:10.1128/IAI.00046-11 21402769 PMC3088142

[55] Kavanaugh JS , Horswill AR . 2016. Impact of environmental cues on staphylococcal quorum sensing and biofilm development. J Biol Chem 291:12556–12564. doi:10.1074/jbc.R116.722710 27129223 PMC4933443

[56] Crosby HA , Schlievert PM , Merriman JA , King JM , Salgado-Pabón W , Horswill AR . 2016. The Staphylococcus aureus global regulator MgrA modulates clumping and virulence by controlling surface protein expression. PLoS Pathog 12:e1005604. doi:10.1371/journal.ppat.1005604 27144398 PMC4856396

[57] Gupta RK , Alba J , Xiong YQ , Bayer AS , Lee CY . 2013. MgrA activates expression of capsule genes, but not the α-toxin gene in experimental Staphylococcus aureus endocarditis. J Infect Dis 208:1841–1848. doi:10.1093/infdis/jit367 23901087 PMC3814835

[58] Jonsson I-M , Lindholm C , Luong TT , Lee CY , Tarkowski A . 2008. mgrA regulates staphylococcal virulence important for induction and progression of septic arthritis and sepsis. Microbes Infect 10:1229–1235. doi:10.1016/j.micinf.2008.07.026 18692591 PMC6734920

[59] Lei MG , Lee CY . 2020. MgrA activates staphylococcal capsule via SigA-dependent promoter. J Bacteriol 203:e00495-20. doi:10.1128/JB.00495-20 33077637 PMC7950413

[60] Luong TT , Dunman PM , Murphy E , Projan SJ , Lee CY . 2006. Transcription profiling of the mgrA regulon in Staphylococcus aureus. J Bacteriol 188:1899–1910. doi:10.1128/JB.188.5.1899-1910.2006 16484201 PMC1426550

[61] Trotonda MP , Tamber S , Memmi G , Cheung AL . 2008. MgrA represses biofilm formation in Staphylococcus aureus. Infect Immun 76:5645–5654. doi:10.1128/IAI.00735-08 18852246 PMC2583562

[62] Chen PR , Bae T , Williams WA , Duguid EM , Rice PA , Schneewind O , He C . 2006. An oxidation-sensing mechanism is used by the global regulator MgrA in Staphylococcus aureus. Nat Chem Biol 2:591–595. doi:10.1038/nchembio820 16980961

[63] Shu X , Shi Y , Huang Y , Yu D , Sun B . 2023. Transcription tuned by S-nitrosylation underlies a mechanism for Staphylococcus aureus to circumvent vancomycin killin. Nat Commun 14:2318. doi:10.1038/s41467-023-37949-0 37085493 PMC10120478

[64] Urbano R , Karlinsey JE , Libby SJ , Doulias P-T , Ischiropoulos H , Warheit-Niemi HI , Liggitt DH , Horswill AR , Fang FC . 2018. Host nitric oxide disrupts microbial cell-to-cell communication to inhibit staphylococcal virulence. Cell Host Microbe 23:594–606. doi:10.1016/j.chom.2018.04.001 29706505 PMC5949146

[65] Pagels M , Fuchs S , Pané-Farré J , Kohler C , Menschner L , Hecker M , McNamarra PJ , Bauer MC , von Wachenfeldt C , Liebeke M , Lalk M , Sander G , von Eiff C , Proctor RA , Engelmann S . 2010. Redox sensing by a Rex-family repressor is involved in the regulation of anaerobic gene expression in Staphylococcus aureus. Mol Microbiol 76:1142–1161. doi:10.1111/j.1365-2958.2010.07105.x 20374494 PMC2883068

[66] Dmitriev A , Chen X , Paluscio E , Stephens AC , Banerjee SK , Vitko NP , Richardson AR , Torres VJ . 2021. The intersection of the Staphylococcus aureus Rex and SrrAB regulons: an example of metabolic evolution that maximizes resistance to immune radicals. mBio 12:e0218821. doi:10.1128/mBio.02188-21 34781744 PMC8593685

[67] Gillaspy AF , Hickmon SG , Skinner RA , Thomas JR , Nelson CL , Smeltzer MS . 1995. Role of the accessory gene regulator (agr) in pathogenesis of staphylococcal osteomyelitis. Infect Immun 63:3373–3380. doi:10.1128/iai.63.9.3373-3380.1995 7642265 PMC173464

[68] Sassi M , Sharma D , Brinsmade SR , Felden B , Augagneur Y . 2015. Genome sequence of the clinical isolate Staphylococcus aureus subsp. aureus strain UAMS-1. Genome Announc 3:e01584-14. doi:10.1128/genomeA.01584-14 25676774 PMC4333674

[69] Jarraud S , Mougel C , Thioulouse J , Lina G , Meugnier H , Forey F , Nesme X , Etienne J , Vandenesch F . 2002. Relationships between Staphylococcus aureus genetic background, virulence factors, agr groups (alleles), and human disease. Infect Immun 70:631–641. doi:10.1128/IAI.70.2.631-641.2002 11796592 PMC127674

[70] Tan L , Huang Y , Shang W , Yang Y , Peng H , Hu Z , Wang Y , Rao Y , Hu Q , Rao X , Hu X , Li M , Chen K , Li S . 2022. Accessory gene regulator (agr) allelic variants in cognate Staphylococcus aureus strain display similar phenotypes. Front Microbiol 13:700894. doi:10.3389/fmicb.2022.700894 35295312 PMC8919982

[71] Montgomery CP , Boyle-Vavra S , Daum RS . 2010. Importance of the global regulators Agr and SaeRS in the pathogenesis of CA-MRSA USA300 infection. PLoS One 5:e15177. doi:10.1371/journal.pone.0015177 21151999 PMC2996312

[72] Larsen FJ , Schiffer TA , Weitzberg E , Lundberg JO . 2012. Regulation of mitochondrial function and energetics by reactive nitrogen oxides. Free Radic Biol Med 53:1919–1928. doi:10.1016/j.freeradbiomed.2012.08.580 22989554

[73] Cleeter MW , Cooper JM , Darley-Usmar VM , Moncada S , Schapira AH . 1994. Reversible inhibition of cytochrome c oxidase, the terminal enzyme of the mitochondrial respiratory chain, by nitric oxide. Implications for neurodegenerative diseases. FEBS Lett 345:50–54. doi:10.1016/0014-5793(94)00424-2 8194600

[74] Giuffrè A , Borisov VB , Arese M , Sarti P , Forte E . 2014. Cytochrome bd oxidase and bacterial tolerance to oxidative and nitrosative stress. Biochim Biophys Acta 1837:1178–1187. doi:10.1016/j.bbabio.2014.01.016 24486503

[75] Giuffrè A , Borisov VB , Mastronicola D , Sarti P , Forte E . 2012. Cytochrome bd oxidase and nitric oxide: from reaction mechanisms to bacterial physiology. FEBS Lett 586:622–629. doi:10.1016/j.febslet.2011.07.035 21821033

[76] Gupta RK , Luong TT , Lee CY . 2015. RNAIII of the Staphylococcus aureus agr system activates global regulator MgrA by stabilizing mRNA. Proc Natl Acad Sci U S A 112:14036–14041. doi:10.1073/pnas.1509251112 26504242 PMC4653210

