# Supplementary material for: Factors impacting the regulation of nos gene expression in Staphylococcus aureus
Source: Microbiol Spectr. 2023 Sep 25;11(5):e01688-23. doi: 10.1128/spectrum.01688-23 (PMC10580903; doi:10.1128/spectrum.01688-23)
Supplement: Supplemental Text S1 — 5' RACE sequences. [file spectrum.01688-23-s0001.docx]

**Supplementary File S1.** 5′ RACE sequence (reverse complement orientation). The mapped TSS is indicated in bold and yellow highlight. Bold headings for each section indicate the template RNA used for cDNA synthesis; primer names are underlined.

**U1 wildtype**

nos-GSP3

>5RACE-1A-nosGSP3B_A08.ab1
NNNNNNANNNNNNNNAGTTNNTTCTTCTGGTATGTNNANNATGGTCCCAGTTTCTTTTATTTCTAGTTCAATNGTCATAGTAAACGTTTATTGATAATTTGCGTTTCATAATGACACTCTTTATACATGTTTTCTATGAAAGCTTGAGCCTCTTTAAATAACATTAACAACACC**T**CCCCCCCCCCCCCCCCCCCNTATTTGGGGGGGGGGNAAAAAN

>5RACE_1-nosGSP-3_A04.ab1
GNNNNNNNNNNANNANTTCTTCTTCTGTATGTGTATATGTCCCAGTTTCTTTTATTTCTAGTTCAATGTCATGTAAACGTTTATTGATAATTTGCGTTTCATAATGACACTCTTTATACATGTTTTCTATGAAAGCTTGAGCCTCTTTAAATAACATTAACAACACC**T**CCCCCCCCCCCCCCCCAAAAAGAAGGGGGGGAAAAAAA

>5RACE_2-nosGSP-3_C04.ab1
GNGNNNNNNNNNNANNNTTCTTCTTCTGTATGTGTATATGTCCCAGTTTCTTTTATTTCTAGTTCAATGTCATGTAAACGTTTATTGATAATTTGCGTTTCATAATGACACTCTTTATACATGTTTTCTATGAAAGCTTGAGCCTCTTTAAATAACATTAACAACACC**T**CCCCCCCCCCCCCCCCATAAAAAAAGGGGGGGAAAAAA

nos-screen

>5race-1A-nos-screen_E06.ab1
NNNNNNNNNNNTGTTTTCTATGAAGNTTNAGCTCTTTAAATAACATTAACAACACC**T**CCCCCCCCCCCCCCCCNNNNTTTNNGGGGGGGGGGAAAAAAAA

>5RACE_1-nos-screen_B04.ab1
NNNNNNNNNNNNNNNTTTTCTNTGANGCTTGAGCCTCTTTAAATAACATTAACAACACC**T**CCCCCCCCCCCCCNNNTTTTNNNNGGGGGGGNAAAAAAA

>5RACE_2-nos-screen_D04.ab1
NNNNNNNNNNNNNTGTTTTCTNTGANGCTTGAGCCTCTTTAAATAACATTAACAACACC**T**CCCCCCCCCCCCCCNNTTTTTTTNNGGGGGGGGAAAAAA

**AH1263**

nos-GSP3

>AH1-nosGSP3_E03.ab1
NNNNNNNNNNNNNNNANTTCTTCTTCTGTATGTGTATATGTCCCAGTTTCTTTTATTTCTAGTTCAATGTCATGTAAACGTTTATTGATAATTTGCGTTTCATAATGACACTCTTTATACATGTTTTCTATGAAAGCTTGAGCCTCTTTAAATAACATTAACAACACC**T**CCCCCCCCCCCCCCCNAAAAANNNNNGGGNNNAAAAA

>AH2-nosGSP3_F03.ab1
GNNNNNNNNNNNNNNNGTTCTTCTTCTGTATGTGTATATNGTCCCAGTTTCTTTTATTTCTAGTTCAATGTCATGTAAACGTTTATTGATAATTTGCGTTTCATAATGACACTCTTTATACATGTTTTCTATGAAAGCTTGAGCCTCTTTAAATAACATTAACAACACC**T**CCCCCCCCCCCCCCCCAAAAATAACCGGGGGAAAAAAAN

nos-screen

>AH1-nos-screen_C04.ab1
NNNNNNNNNNNNNNNNNTTTCTATGANGCTTGAGCCTCTTTAAATAACATTAACAACACC**T**CCCCCCCCCCCNNNNNNNNNNNNNGGGGGGGGNAAAAN

>AH2-nos-screen_D04.ab1
NNNNNNNNNNNNNNTGNTTTCNNTGAAGCTTGAGCCTCTTTAAATAACATTAACAACACC**T**CCCCCCCCCCCCCNTTTNTTNNNNNNGGGGGGNAAAANN

**U1 pJBnos1**

lacZ-GSP3

>nos1-lacZ-GSP3_C08.ab1
NNNNNNNNNNNNACCNTGATTCTGGACTGCTTCAGGTGCTGGAAACCATGCAAAACGCCATTCACCATTTAATGAACGTAATTGTTGTGATGGACGATCTGTACGTGCTTCTTCTGAATTACGCCATGATGCAAATGGTGGATGTGCTGCTAAACGATTTAATTGTGTAACACCTGGATTTTCCCAATCACGACGTTGTAAAACAACTGCTAATGAATCTGTAATCATTGTCATTCTAGACATTAACAACACC**T**CCCCCCCCCCCCCCCCCCCATAAAAGGGGGGGGGAAAAAAAA

>nos1-lacZ-GSP3_D08.ab1
NNNNNNNNNNNNNNNTGATTCTGGAACTGCTTCAGGTGCTGGAAACCATGCAAAACGCCATTCACCATTTAATGAACGTAATTGTTGTGATGGACGATCTGTACGTGCTTCTTCTGAATTACGCCATGATGCAAATGGTGGATGTGCTGCTAAACGATTTAATTGTGTAACACCTGGATTTTCCCAATCACGACGTTGTAAAACAACTGCTAATGAATCTGTAATCATTGTCATTCTAGACATTAACAACACC**T**CCCCCCCCCCCCCCCCCCCAAAAAAGGGGGGGGGAAAAAAAA

lacZ-screen

>nos1-lacZ-screen_G08.ab1
NNNNNNNNNNATTNTNTTCTAGACATTAACAACACC**T**CCCCCCCCCCCCCCCCCNNTTTTTNNNGGGGNNNNNNNNAAAN

>nos1-lacZ-screen_H08.ab1
NNNNNNNNNTNTTNTNNTTCTAGACATTAACAACACC**T**CCCCCCCCCCCCCCCCCNNNTNNNNNNANGGNNNNNNNNNNAN

**U1 pJBnos2**

lacZ-GSP3

>nos2-lacZ-GSP3_A05.ab1
NNNNNANNNNNNNNNNNNNNNNNNNNANNNNNNNNNTGGCTGGAAACCATGCAAAACGCCATTCACCATTTAATGAACGTAATTGTTGTGATGGACGATCTGTACGTGCTTCTTCTGAATTACGCCATGATGCAAATGGTGGATGTGCTGCTAAACCATTTAATTGTGTAACACCTGGATTTTCCCAATCACGACGTTGTAAAACAACTGCTAATGAATCTGTAATCATTGTCATTCTAGACATATGTTTTTCCTCCTTATAAAGTTAATCAGTCGACCCCGGGGGATCCATAGTCT**A**CCCCCCCCCCCCCCCCCTTAAATTCCACCGGGGNAAAAAA

>nos2-lacZ-GSP3_C05.ab1
NNNNNNNNNNNANNNNNANNNNNGNNAANNNNCNNNAGNNNNNNNNAAACCATGCAAAACGCCATTCACCATTTAATGAACGTAATTGTTGTGATGGACGATCTGTACGTGCTTCTTCTGAATTACGCCATGATGCAAATGGTGGATGTGCTGCTAAACCATTTAATTGTGTAACACCTGGATTTTCCCAATCACGACGTTGTAAAACAACTGCTAATGAATCTGTAATCATTGTCATTCTAGACATATGTTTTTCCTCCTTATAAAGTTAATCAGTCGACCCCGGGGGATCCATAGTCT**A**CCCCCCCCCCCCCCCAAAAATTNNGNGGGAAAAAA

lacZ-screen

>nos2-lacZ-screen_B05.ab1
NNNNNNNNNNNNTTGTCTTCTAGACATATGTTTTTCCTCCTTATAAAGTTAATCAGTCGACCCCGGGGGATCCATAGTCT**A**CCCCCCCCCCCCCCCAAATTTCCNCNGGGANNNN

>nos2-lacZ-screen_D05.ab1
NNNNNNNNNNNNTTGTCATTCTAGACATATGTTTTTCCTCCTTATAAAGTTAATCAGTCGACCCCGGGGGATCCATAGTCT**A**CCCCCCCCCCCCCCCCAAATNNNCCCGNNNNAAANA
