# Supplementary material for: Factors impacting the regulation of nos gene expression in Staphylococcus aureus
Source: Microbiol Spectr. 2023 Sep 25;11(5):e01688-23. doi: 10.1128/spectrum.01688-23 (PMC10580903; doi:10.1128/spectrum.01688-23)
Supplement: Supplemental Figure S1 [file spectrum.01688-23-s0002.pdf]

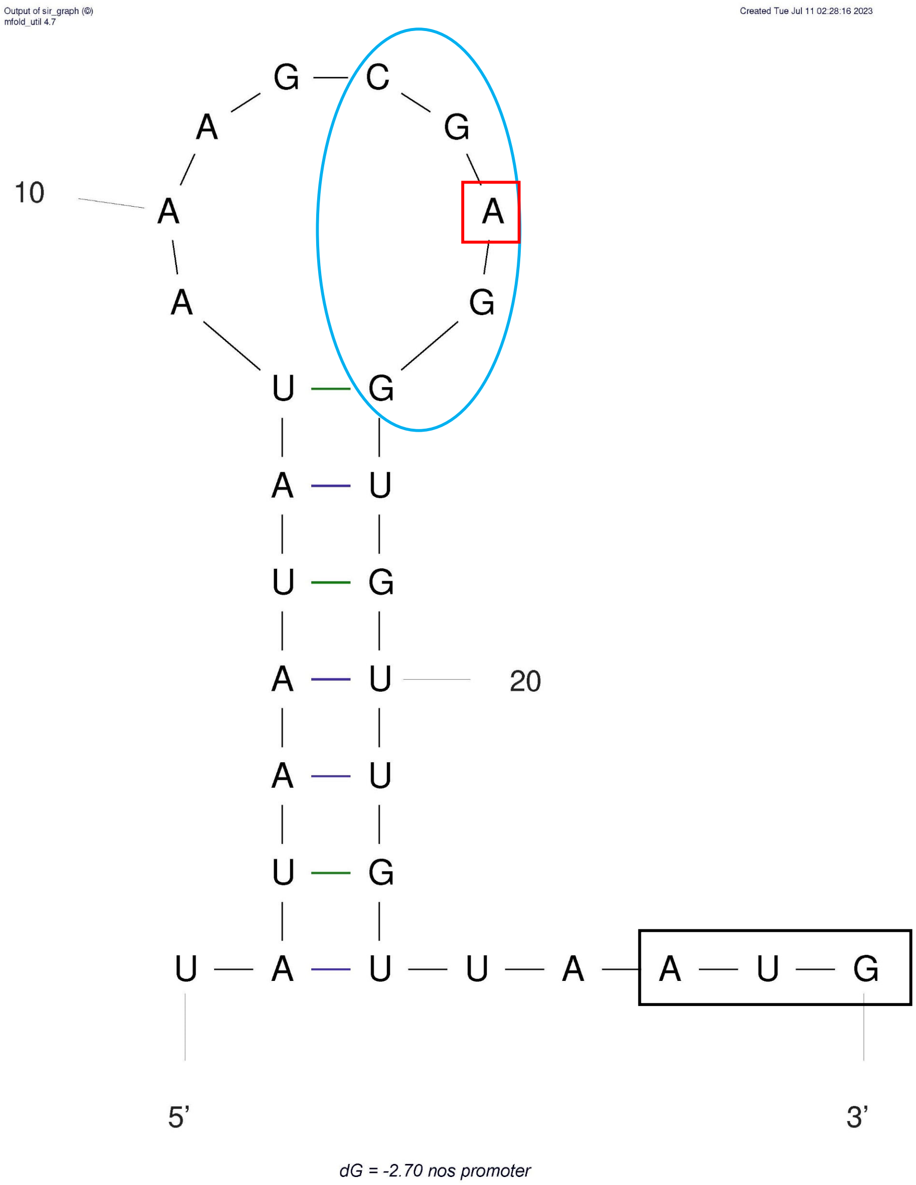

Supplemental Figure S1. Analysis of *nos* promoter region RNA secondary structure using mFold (37°C folding temperature, 53.2°C predicted melting temperature). The mFold predicted structure of the 25 bp sequence upstream to the predicted *nos* ATG start codon (outlined in black box) is shown. The putative SD sequence is outlined in blue, and the TSS-1 adenine nucleotide is outlined in red.
