# Supplementary material for: Factors impacting the regulation of nos gene expression in Staphylococcus aureus
Source: Microbiol Spectr. 2023 Sep 25;11(5):e01688-23. doi: 10.1128/spectrum.01688-23 (PMC10580903; doi:10.1128/spectrum.01688-23)
Supplement: Supplemental Tables S1 and S2 — Supplemental Table S1 - strains and plasmids. Table S2 - primers. [file spectrum.01688-23-s0003.docx]

Table S1. Bacterial strains and plasmids used in this study

| Strain or plasmid | Description | Source |
| --- | --- | --- |
| *Escherichia coli* |  |  |
| DH5α | Host strain for construction of recombinant plasmids | (1) |
|  |  |  |
| *Staphylococcus aureus* |  |  |
| RN4220 | Easily transformable restriction-deficient strain | (2) |
| UAMS-1 | Wildtype osteomyelitis clinical isolate | (3) |
| KB6004 | UAMS-1 Δ*srrAB* mutant | (4) |
| KR1300 | UAMS-1 Δ*agr* mutant | This study |
| KR6300 | UAMS-1 Δ*srrAB* Δ*agr* mutant | This study |
| *rex* | UAMS-1 *rex*::*kan* insertion mutant | This study |
| *mgrA* | UAMS-1 Δ*mgrA* mutant | (5) |
| AH1263 | Erm-sensitive LAC (wildtype CA-MRSA USA300) | (6) |
| JLB316 | AH1263 Δ*agr* mutant | This study |
|  |  |  |
| Plasmids |  |  |
| pCR-Blunt | Cloning plasmid; Kan^R^ | Invitrogen |
| pJB38 | Temperature-sensitive allelic exchange plasmid; Cm^R^ | (7) |
| pMP9 | *agr* deletion allele in pJB38 | This study |
| pJF102 | Δ*rex::kan* in shuttle vector pBT2ts | (8) |
| pJB185 | Promoterless codon-optimized *lacZ*; Cm^R^ | (9) |
| pJBnos1 | *nos-lacZ* fusion in pJB185; *nos* predicted start codon and 508 bp upstream region, including the putative Shine-Dalgarno (SD) sequence | This study |
| pJBnos2 | *nos-lacZ* fusion in pJB185; 500 bp region upstream to the predicted *nos* SD sequence; does not include SD or *nos* start codon (translation driven by non-native SD sequence and translation enhancer region present upstream of *lacZ* in pJB185) | This study |

Table S2. Primers used in this study

| Primer | Sequence (5′-3′) | Purpose | Source |
| --- | --- | --- | --- |
| nos-GSP1  nos-GSP2  nos-GSP3  nos-screen | TCTTTTTCAGCAGGATCACCAC  CGATGCTTCGTCAGTAACATCT  GCAACGATTTGAATTACGCCAA  CGTTTATTGATAATTTGCGTTTCA | *nos* 5′ RACE | This study |
|  |  |  |  |
| lacZ-GSP1  lacZ-GSP2  lacZ-GSP3  lacZ-screen | TGGTAAACGTGAATCTTGACCA  TTCTGTTGGAACAAAAGGTGGA  GCACAACAACTGTATCTGCTTC  ACGACGTTGTAAAACAACTGCT | *lacZ* 5′ RACE | This study |
|  |  |  |  |
| nos_F  nos_R | cccgaattcTACCAAAGCATAATTCGCCT  ccctctagaCATTAACAACACCTCGCTTT | pJBnos1 | This study |
|  |  |  |  |
| nosX_F  nosX_R | cccgaattcTAACAATGGTTCGTTACCAAAG  cccggatccATAGTCTACATTATTAAAATACT | pJBnos2 | This study |
|  |  |  |  |
| colacZ_R | ACCACACATTTGCACTTCAGC | *nos-lacZ* and *cid-lacZ* PCR confirmation | This study |
|  |  |  |  |
| nos4-F  nos4-R | TATGGTGCTAAAATGGCTTG  ACGATGCTTCGTCAGTAACA | *nos* qPCR | (10) |
|  |  |  |  |
| sigA-F  sigA-R | CAAGCAATCACTCGTGCAAT  GGTGCTGGATCTCGACCTAA | *sigA* qPCR (housekeeping control) | (10) |
|  |  |  |  |
| agr_5′F  agr_5′R | cccgaattcAATTTCTCTATGTTGGGGCC  cccctcgagAATAAGTATGGTCGTGAGCC | UAMS-1 Δ*agr* mutant creation | This study |
| agr_3′F | cccctcgagGTTGCAGCGATGGATTTTAT |  |  |
| agr_3′R | cccgtcgacACAGTGTAGGAAACAAGTTGA |  |  |
|  |  |  |  |
| NS24 | cagggtaccgctagctttttaattttattaacaaaattaaatatgacgc | AH1263 Δ*agr* mutant creation | This study |
| JBKU123 | caagtcgacGTATTCATGATGAAAATGAAGCCA TTCAATC |  |  |
| JBKU120 | cagaattctgaagtagatgtagtcgttttacctgaaatg |  |  |
| JBKU121 | cagctagcAAAATATATGAATAAGTCTAATGTTGGAAAAGG |  |  |
|  |  |  |  |
| agr_up_F  agr_down_R | GTCAACTCAAACAAATGCCA  TGCAATGTAGCTTGTACAGT | Δ*agr* mutant confirmation | This study |

Lowercase letters in primer sequence represent non-native sequence nucleotides.

References:

1. Liebeke M, Lalk M. 2014. *Staphylococcus aureus* metabolic response to changing environmental conditions - a metabolomics perspective. Int J Med Microbiol 304:222-9.

2. Somerville GA, Proctor RA. 2009. At the crossroads of bacterial metabolism and virulence factor synthesis in Staphylococci. Microbiol Mol Biol Rev 73:233-48.

3. Lee DS, Burd H, Liu J, Almaas E, Wiest O, Barabási AL, Oltvai ZN, Kapatral V. 2009. Comparative genome-scale metabolic reconstruction and flux balance analysis of multiple *Staphylococcus aureus* genomes identify novel antimicrobial drug targets. J Bacteriol 191:4015-24.

4. Vaish M, Singh VK. 2013. Antioxidant Functions of Nitric Oxide Synthase in a Methicillin Sensitive *Staphylococcus aureus*. International Journal of Microbiology.

5. Favazzo LJ, Gill AL, Farnsworth CW, Mooney RA, Gill SR. 2019. The Response of nor and nos Contributes to Staphylococcus aureus Virulence and Metabolism. J Bacteriol 201.

6. Boles BR, Thoendel M, Roth AJ, Horswill AR. 2010. Identification of genes involved in polysaccharide-independent *Staphylococcus aureus* biofilm formation. PLoS One 5:e10146.

7. Bose JL, Fey PD, Bayles KW. 2013. Genetic tools to enhance the study of gene function and regulation in Staphylococcus aureus. Appl Environ Microbiol 79:2218-24.

8. Crooke AK, Fuller JR, Obrist MW, Tomkovich SE, Vitko NP, Richardson AR. 2013. CcpA-independent glucose regulation of lactate dehydrogenase 1 in *Staphylococcus aureus*. PLoS One 8:e54293.

9. Krute CN, Rice KC, Bose JL. 2017. VfrB Is a Key Activator of the *Staphylococcus aureus* SaeRS Two-Component System. J Bacteriol 199.

10. Sapp AM, Mogen AB, Almand EA, Rivera FE, Shaw LN, Richardson AR, Rice KC. 2014. Contribution of the nos-pdt Operon to Virulence Phenotypes in Methicillin-Sensitive Staphylococcus aureus. PLoS One 9.
